# Incidental finding of a gastric schwannoma: a case report

**DOI:** 10.1093/jscr/rjab509

**Published:** 2021-11-12

**Authors:** Nelimar Cruz Centeno, Albert Suarez Dominguez, Pablo Mojica Mañosa, Victor P Carlo

**Affiliations:** Department of General Surgery, University of Puerto Rico, Medical Sciences Campus, San Juan, Puerto Rico; Department of General Surgery, University of Puerto Rico, Medical Sciences Campus, San Juan, Puerto Rico; Department of General Surgery, University of Puerto Rico, Medical Sciences Campus, San Juan, Puerto Rico; Department of Anatomic Pathology, Auxilio Mutuo Hospital, San Juan, Puerto Rico

## Abstract

Gastric schwannomas are rare peripheral nerve sheath tumors which are usually found incidentally while undergoing workup for other conditions. Despite their benign nature, they require surgical resection with negative margins. It is important to differentiate gastric schwannomas from gastrointestinal stromal tumors prior to surgical excision, as this can alter the recommended surgical plan. This can be achieved with endoscopic ultrasound and fine needle aspiration with analysis of the sampled tissue using immunohistochemical stains. We present the case of a 68-year-old female patient with an incidental finding of a gastric fundus schwannoma. Laparoscopic gastric wedge resection was performed with complete excision of the tumor and negative margins. Pathology was confirmed with immunohistochemical stains positive for S-100 and negative for CD117 and DOG1. Post-operative recovery was uneventful without tumor recurrence.

## INTRODUCTION

A schwannoma is a benign tumor arising from Schwann cells, which are found in peripheral nerve sheaths. The most common locations of this tumor include the head, neck and extremities. Although uncommon, they have been reported in the gastrointestinal (GI) tract, with the stomach being the most common location (60–70% of cases) [1, 2]. Gastric schwannomas are rare and have been reported to represent ~0.2% of all gastric tumors [3, 4]. They can be difficult to differentiate from other stomach tumors, such as gastrointestinal stromal tumors (GIST), sarcomas and leiomyomas based on imaging alone. Pathology and immunohistochemistry characterization is necessary for final diagnosis. Most schwannomas are benign and asymptomatic. However, malignant transformation can occur, and if large enough, they can compress nearby structures, cause ulceration of the gastric mucosa and bleeding. Therefore, the recommended management is complete surgical excision. We present the case of a 68-year-old female patient with incidental finding of an asymptomatic stomach mass that was confirmed to be a gastric schwannoma after surgical excision.

## CASE REPORT

**Figure 1:**
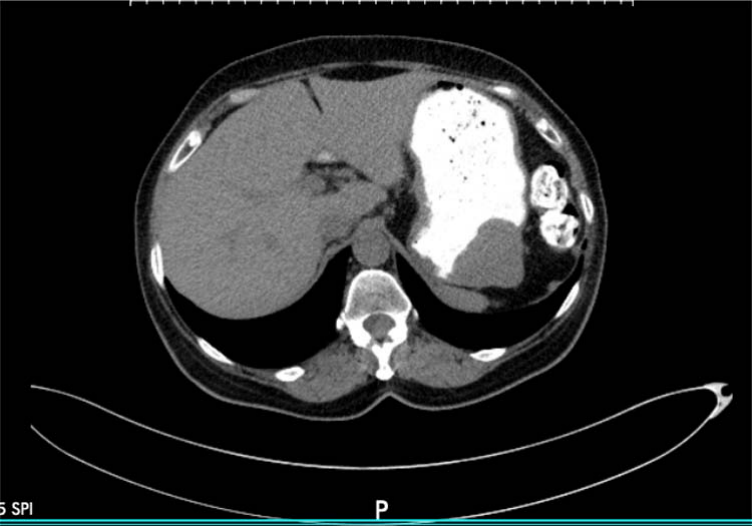
Axial view of the abdominopelvic CT scan with oral contrast showing a well-circumscribed gastric soft tissue attenuation. P: Posterior.

We present the case of a 68-year-old female patient with a history of hypertension and hypothyroidism who was being evaluated at the surgery clinics due to complains of a left inguinal bulging concerning for a hernia. She denied abdominal pain, nausea, vomiting or any other symptoms related to the GI tract. Abdominopelvic computed tomography (CT) scan with oral contrast was ordered for further evaluation. Imaging results were remarkable for a well-circumscribed soft tissue submucosal mass in the gastric body, measuring 4.4 × 4.3 × 3.2 cm (Fig. 1). There was no evidence of transmural invasion or lymphadenopathy, and the rest of the GI tract was within normal limits. Subsequently, the patient underwent esophagogastroduodenoscopy with endoscopic ultrasound (EUS) and fine needle aspiration (FNA). EUS was remarkable for a hypoechoic upper stomach lesion, arising from the muscularis propia of the gastric wall and measuring 4 cm. Based on EUS results, the lesion was suspicious for a GIST. The first FNA was non diagnostic, and another biopsy with tattooing of the lesion was performed. The second FNA was positive for a spindle cell neoplasm, consistent with schwannoma.

**Figure 2:**
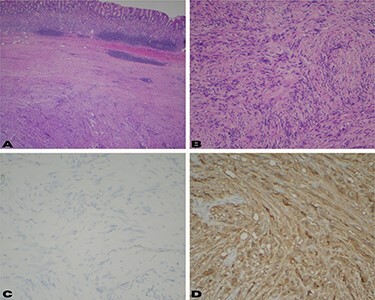
(**A**) Low power view shows a well-circumscribed submucosal mass composed of spindle cells with variable cellularity ranging from hypocellular to moderately cellular; (**B**) higher magnification at 10× shows spindle cells with tapered ends and no significant cytologic atypia; occasional areas show nuclear palisades; (**C**) CD117 and DOG-1 immunohistochemical stains are negative in the surgical specimen; (**D**) immunohistochemical stain for S-100 shows strong diffuse cytoplasmic staining.

The patient was scheduled to undergo laparoscopic resection of the mass. During surgery, the previously tattooed lesion was identified at the gastric fundus and a wedge resection of that segment was performed, achieving complete resection of the tumor. Pathology confirmed the diagnosis of a schwannoma (Fig. 2). Immunohistochemical stains were positive for S-100 (Fig. 2D) and were negative for CD117 and DOG1 (Fig. 2C). The specimen measured 4.2 × 4 × 4.5 cm and had a 5-cm margin that was free of disease. The patient recovered well after surgery with no complications and was discharged home on post-operative day two.

## DISCUSSION

Gastric schwannomas are rare benign stomach tumors arising from Scwhann cells in the nerve sheath of Auerbach’s or Meissner’s plexus [5, 6]. As in this case, this diagnosis has been predominantly found in females between their fifth and eighth decades of life [1, 5, 6]. Most patients are asymptomatic, and the tumor is often found incidentally on abdominal CT scan or endoscopy. However, symptoms can include abdominal pain, upper GI bleeding, decreased appetite, dyspepsia, nausea or vomiting.

Gastric schwannomas can be mistaken for other neoplasms based on imaging and endoscopic findings. They are commonly misdiagnosed for GIST tumors, which can be malignant in 10–30% of cases [5–8]. Gastric schwannomas are described as having well-demarcated homogenous density on CT scan [9]. By contrast, GIST are defined as a heterogeneous enhancing lesion with low attenuation secondary to necrosis or hemorrhage [10]. It is important to differentiate schwannomas from other gastric tumors with higher malignant potential, as this will dictate surgical approach and extent of resection. In this case, both CT scan and EUS reports were suggestive of a GIST despite the final pathology revealed a schwannoma.

EUS with FNA is used for pre-operative diagnosis of submucosal gastric tumors and surgical planning. FNA is a low-risk procedure that has been shown to have a diagnostic accuracy of 62–93% for subepithelial lesions of the GI tract [7]. Both schwannomas and GIST have a histological spindle cell appearance (Fig. 2B). Therefore, immunohistochemical staining is of paramount importance to differentiate between the two. GIST tumors stain positive for CD117 antigen as well as CD34, whereas schwannomas are negative for these markers and positive for S-100. S-100 is a protein derived from neural crest cells and is the most reliable indicator for the diagnosis of a schwannoma [9]. It is very important to make this distinction prior to surgical intervention since the management of a GIST varies with tumor size and mitotic rate [11].

Gastric schwannomas are rarely malignant. Most of them can be managed with excision of the part of the stomach containing the tumor without lymph node sampling because they rarely metastasize to the lymph nodes [9]. They are commonly located in the gastric body (59%); however, this tumor was found in the gastric fundus which has been reported to be the location in ~12–18% of gastric schwannomas [5, 6, 9]. This location allowed us to perform a wedge resection of the involved gastric segment, leaving most of the stomach intact. Sometimes partial, subtotal or total gastrectomy is required if excision cannot be achieved by wedge resection. In view of the low malignant potential, there is no standard margin of resection. However, it is generally accepted that surgical margins should be free of disease to prevent recurrence. This can be achieved successfully with laparoscopic resection.

CT scan follow-up is not recommended due to the benign nature of the neoplasm. There are reports of a disease-free survival of >36 months after undergoing surgery with negative margins [5]. Once the diagnosis has been done, the management of a gastric schwannoma is straightforward. What poses a challenge to the surgeon is making the correct diagnosis. Thus, it is important to keep in mind this diagnosis when dealing with submucosal gastric tumors. Pre-operative tumor histologic examination should be attained prior to surgery to ensure the patient receives the correct surgical approach based on the type of neoplasm and its malignant potential.

## CONFLICT OF INTEREST STATEMENT

None declared.

## FUNDING

The authors state that there are no sources of funding to disclose.
